# Antiparasitic Activity of Enterocin M and Durancin-like from Beneficial *Enterococci* in Mice Experimentally Infected with *Trichinella spiralis*

**DOI:** 10.3390/microorganisms12050923

**Published:** 2024-05-01

**Authors:** Miroslava Petrová, Zuzana Hurníková, Andrea Lauková, Emília Dvorožňáková

**Affiliations:** 1 Institute of Parasitology, Slovak Academy of Sciences, 040 01 Kosice, Slovakia; vargovam@saske.sk (M.P.); hurnikz@saske.sk (Z.H.); 2 Institute of Animal Physiology, Centre of Biosciences, Slovak Academy of Sciences, 040 01 Kosice, Slovakia; laukova@saske.sk

**Keywords:** Enterocin M, Durancin-like, *Enterococcus*, *Trichinella spiralis*

## Abstract

Beneficial/probiotic strains protect the host from pathogens by competitive displacement and production of antibacterial substances, i.e., bacteriocins. The antiparasitic potential of bacteriocins/enterocins and their producing strains in experimental murine trichinellosis were tested as a new therapeutic strategy. Enterocin M and Durancin-like and their producers *Enterococcus faecium* CCM8558 and *Enterococcus durans* ED26E/7 were administered daily to mice that were challenged with *Trichinella spiralis*. Our study confirmed the antiparasitic effect of enterocins/enterococci, which reduced the number of adults in the intestine (Enterocin M—43.8%, *E. faecium* CCM8558—54.5%, Durancin-like—16.4%, *E. durans* ED26E/7—35.7%), suppressed the *Trichinella* reproductive capacity ex vivo (Enterocin M—61%, *E. faecium* CCM8558—74%, Durancin-like—38%, *E. durans* ED26E/7—66%), and reduced the number of muscle larvae (Enterocin M—39.6%, *E. faecium* CCM8558—55.7%, Durancin-like—15%, *E. durans* ED26E/7—36.3%). The direct effect of enterocins on *Trichinella* fecundity was documented by an in vitro test in which Durancin-like showed a comparable reducing effect to Enterocin M (40–60%) in contrast to the ex vivo test. The reducing activity of *T.spiralis* infection induced by Enterocin M was comparable to its strain *E. faecium* CCM8558; Durancin-like showed lower antiparasitic activity than its producer *E. durans* ED26E/7.

## 1. Introduction

Trichinellosis is a worldwide zoonosis caused by infectious larvae of *Trichinella* spp. from a broad range of hosts (domestic animals, wildlife) and the third most serious food-borne parasitosis with an important public health risk and economic losses [1,2]. Available chemotherapy (benzimidazoles) is effective only against adult stages in the gut, but it does not work against migrating and encapsulated muscle larvae [3]. Therefore, it is necessary to develop effective alternatives for controlling trichinellosis, such as by harnessing the antiparasitic potential of plants, natural proteins, and probiotics [4,5,6,7,8].

Probiotic/beneficial bacteria can successfully reduce the pathogenicity of many parasites [9,10]. Several works described an active role of probiotics in trichinellosis control [8,11,12,13,14,15]. Probiotics’ mode of action consists of competitive elimination of pathogens, enhancement of the gut epithelial barrier, secretion of active molecules, and immunomodulation [9]. Recent studies confirmed that the whole organism of probiotic bacteria can be exchanged by their metabolites (proteins, lipids, polysaccharides) to achieve their immunomodulatory activity [16]. 

Lactic acid bacteria (LAB) produce bacteriocins. Bacteriocins are extracellular antimicrobial protein molecules synthesized on ribosomes. They are relatively small cationic molecules (30 to 60 amino acids), differing in their mode of action, molecular weight, genetic origin, and biochemical properties [17]. The large genus *Enterococcus* represents the third most numerous genus of LAB bacteria, after the genera *Lactobacillus* and *Streptococcus*. Among enterococci, *Enterococcus faecium* is the most frequent producer of bacteriocins, i.e., enterocins [18]. The mechanism of action of enterocins consists of disrupting the cell membrane permeability and integrity, resulting in the target cell death [19]. The importance of bacteriocins lies in maintaining the homeostasis of the organism, but they are also used against viral infections and in the prevention of inflammatory diseases or systemic infections [20]. Currently, bacteriocins are a very promising natural alternative to antibiotics and chemical preservatives [20]. Several studies have described the inhibitory activity of bacteriocins of LAB to various viruses [21,22,23]. The antiparasitic action of bacteriocins has been recorded only in leishmaniasis [24] and in trichinellosis by our team [25]. 

The study of the antiparasitic properties of enterocins must take into account a number of factors that affect the specific activity of enterocins: dosage scheme, animal model, and enterocins’ functionality in in vitro versus in vivo studies. Our study was focused on a prophylactic and controlling treatment. The enterocins/strains were started one week before the parasite challenge but also continued during the development of the infection, every day throughout the experiment. The intestinal phase in *T. spiralis* life cycle [1] is relatively short considering the time required for intestinal colonization (about 5 days). *T. spiralis* larvae L1 molt four times and become sexually mature within 30 h after penetration of the mucosa of the small intestine. Larvae develop into sexually mature adults within 2 days and then copulate. Females are viviparous and release newborn larvae 5–7 days after infection [1]. Therefore, the therapy was started before the infection as a prophylactic measure, so that the reproductive activity of the parasite could be affected. 

The present study aimed to evaluate the antiparasitic activity of enterocins Enterocin M and Durancin-like in comparison to their producing strains *E. faecium* CCM8558 and *E. durans* ED26E/7 against *T. spiralis* infection in an experimental murine model. 

## 2. Materials and Methods

### 2.1. Ethical Approval

Experimental animals were kept in compliance with Slovak ethical rules according to the Guidelines for Care and Use of Laboratory Animals of the Institute of Parasitology SAS, and the experimental protocol was approved by the State Veterinary and Food Administration of the Slovak Republic (No. Ro-1604/19-221/3).

### 2.2. Enterocins and Their-Producing Strains

The beneficial strains *E. faecium* AL41 = CCM 8558 and *E. durans* ED26E/7 (the Centre of Biosciences of the Slovak Academy of Sciences, Institute of Animal Physiology, Košice, Slovakia) were prepared and evaluated according to the EFSA rules. After the strains’ cultivation, the bacterial cells were adjusted to a concentration of 10^9^ colony-forming units per mL (CFU/mL). *E. faecium* CCM 8558 is an environmental isolate and deposed in the Czech Culture Collection of Microorganisms, Brno, Czech Republic [25]. *Enterococcus durans* ED26E/7 was isolated from ewe’s milk lump cheese [26].

Enterocin M is a new type of antimicrobial substance—a bacteriocin—which belongs to the IIa class of enterocins [27] and can be allotted to postbiotics [28]. It is produced by the strain *E. faecium* CCM8558. The Enterocin M was isolated and characterized by Mareková et al. [29]. It is a thermo-stable, small peptide (molecular weight 4628 Da) with a broad antimicrobial spectrum. For treatment, a precipitate (partially purified substance) was prepared as follows: The producer strain was grown in an appropriate volume of MRS broth (Merck, Darmstadt, Germany) for 18 h at 37 °C. Then it was centrifuged (10,000× *g* for 30 min at 4 °C). The supernatant (pH 5.5) was precipitated with ammonium sulphate (40% saturation) at laboratory temperature for 1 h by stirring. Then it was centrifuged again (10,000× *g*, 30 min), and pellets were re-suspended in 10 mM phosphate buffer (pH 6.5) in minimum volume. This precipitate was checked for its inhibitory activity (51,200 AU/mL) against the principal indicator strain *E. avium* EA5, and the concentration of 2 mg/mL was used for treatment. 

Durancin-like is produced by the food-derived strain *E. durans* ED26E/7. It is also a small peptide with a broad antimicrobial spectrum and belongs to the group of pediocin-like enterocins (group II.a) [27]. The partially purified substance for treatment was prepared as follows [26]: The strain was grown in MRS broth (Merck) in an appropriate volume for 18 h at 37 °C. Then it was centrifuged (10,000× *g* for 30 min at 4 °C). The supernatant (pH 4.2) was precipitated with ammonium sulphate (80% saturation). After precipitation, it was centrifuged (10,000× *g* for 30 min at 4 °C), and pellets of the precipitate were re-suspended in phosphate buffer (pH 6.5) in minimum volume. Then inhibitory activity was checked using the agar spot test [30] and reached inhibitory activity (25,600 AU/mL), and the concentration of 2 mg/mL was used for treatment.

### 2.3. Experimental Design

**Figure 1:**
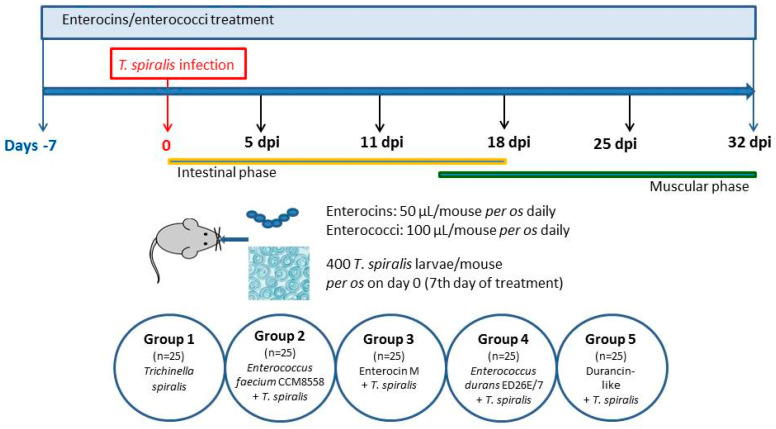
Scheme of the experiment.

The experiment (Figure 1) was carried out on 125 BALB/c mice (male, 8 weeks old, weight of 18–20 g) supplied from VELAZ (Prague, Czech Republic). Animal housing met the following conditions: room temperature 22–24 °C, humidity 56%, 12 h light/dark regime, a commercial diet, and water ad libitum. There were 5 groups of animals in the experiment: Group 1 (*n* = 25)—*T. spiralis* infection, without treatment; Group 2 (*n* = 25)—*E. faecium* CCM8558 + *T. spiralis*; Group 3 (*n* = 25)—Enterocin M + *T. spiralis*; Group 4 (*n* = 25)—*E. durans* ED26E/7 + *T. spiralis*; Group 5 (*n* = 25)—Durancin-like + *T. spiralis. Enterococcus* strains (100 µL of 10^9^ CFU/mL) and enterocins (50 µL of 2 mg/mL) were administered daily per os by an oral gavage throughout the experiment. No mice treated with enterocins/enterococci died before reaching the end of the experiment. Mice were infected with 400 *T. spiralis* larvae L1 (reference isolate *T. spiralis* ISS 004) on the 7th day of enterocins/enterococci treatment. Samples of the small intestines and muscles were taken from the animals (5 mice/group) on the following days post infection (dpi): 5, 11, 18, 25, and 32.

### 2.4. Parasite Burden 

The intestinal phase of trichinellosis was evaluated according to the number of worms isolated from the small intestine (5, 11, and 18 dpi). Small pieces of the gut (5–10 cm) were incubated on sieves in conical glasses in 0.9% saline at 37 °C overnight. Then, the worms in the sediment were counted under a stereomicroscope (60× magnifications, Leica S8APO, Leica Microsystems, Wetzlar, Germany). 

The muscular phase of trichinellosis was evaluated according to the number of larvae isolated from the muscles of the host (18, 25, and 32 dpi). The whole skinned, eviscerated, ground mouse carcasses were digested according to Kapel and Gamble [31]. Larvae from the sediment were counted under a stereomicroscope (40× magnifications, Leica S8APO, Leica Microsystems, Germany). The results were expressed as the number of larvae recovered per mouse. 

Parasite reduction rate was expressed as a percentage reduction (R) and was calculated as $R=\frac{I-T}{I}\times 100\%$; I—the numbers of adults/larvae per mice recovered from the untreated infected group (group 1); T—the numbers of adults/larvae per mice recovered from infected groups treated with enterocins/enterococci (groups 2, 3, 4, or 5).

### 2.5. T. spiralis Female’s Fecundity Ex Vivo and In Vitro

Both tests included 14 mice (BALB/c) in the experiment as follows: Group 1 (*n* = 6)—*T. spiralis* + no treatment; Group 2 (*n* = 2)—*E. faecium* CCM8558 + *T. spiralis*; Group 3 (*n* = 2)—Enterocin M + *T. spiralis*; Group 4 (*n* = 2)—*E. durans* ED26E/7 + *T. spiralis*; Group 5 (*n* = 2)—Durancin-like + *T. spiralis.* Mice were treated with enterococcis (100 µL of 10^9^ CFU/mL) and enterocins (50 µL of 2 mg/mL) daily per os throughout the experiment and infected with *T. spiralis* (400 larvae L1) on the 7th day of treatment. Ex vivo fecundity test: adult females of *T*. *spiralis* were isolated from the gut (2 mice/group) on 5 dpi. In vitro fecundity test: adult females of *T*. *spiralis* were isolated from the gut (4 mice/Group 1) on 5 dpi. The live *T*. *spiralis* females were isolated according to Cabaj [32]. 

Ex vivo fecundity test: The females isolated from mice treated with enterocins/enterococci and infected (2 mice/group and 5 females/mouse) were washed with RPMI 1640 medium (Sigma-Aldrich, Darmstadt, Germany) and placed individually into wells of a 24-well tissue culture plate with 500 µL of RPMI medium + 3% fetal bovine serum + 100 U/mL penicillin + 100 U/mL streptomycin (all Sigma-Aldrich, Germany). The females were incubated at 37 °C in 5% CO_2_ for 20 h. Newborn larvae (NBL) were counted per one female under an inverted microscope (60× magnifications, Leica DM IL LED, Leica Microsystems, Germany). Reduction in fecundity (number of NBL per female) was expressed as percentage reduction (R) and was calculated as $R=\frac{I-T}{I}\times 100\%$; I—number of NBL given by females from the untreated infected group (Group 1); T—number of NBL given by females from infected groups treated with enterocins/enterococci (Groups 2, 3, 4, or 5).

In vitro fecundity test: The subjects were the females isolated from untreated infected mice (Group 1, *n* = 4) on 5 dpi. A total of (30 females/mouse) were incubated in 500 µL of RPMI 1640 medium + 3% fetal bovine serum (both Sigma-Aldrich, Germany) with an examined enterocin/*Enterococcus* for 20 h at 37 °C in 5% CO_2_. Enterocin M and Durancin-like were diluted in the cultivation medium to 1:10^2^, 1:10^4^, 1:10^6^, 1:10^8^. *E. faecium* CCM8558 and *E. durans* ED26E/7 strains were diluted to concentrations of 10^7^, 10^5^, 10^3^, and 10^1^ CFU/mL). The wells containing only cultivation medium and without enterocin/*Enterococcus* were the control. NBL were counted per one female under an inverted microscope (60× magnifications, Leica DM IL LED, Leica Microsystems, Germany). Reduction in fecundity (number of NBL per female) was expressed as percentage reduction (R) and was calculated as $R=\frac{I-T}{I}\times 100\%$; I—number of NBL from untreated females; T—number of NBL from females treated with enterocins/enterococci.

### 2.6. Statistical Analysis

The data were presented as the mean value ± standard deviation (SD). One-way ANOVA and post hoc Tukey’s test in STATISTICA (data analysis software system), version 12 (StatSoft, Inc., Tulsa, OK, USA, 2013, www.statsoft.com, accessed on 10 August 2022) were used. Statistical significance was defined at *p* < 0.05.

## 3. Results

### 3.1. Parasite Burden

**Figure 2:**
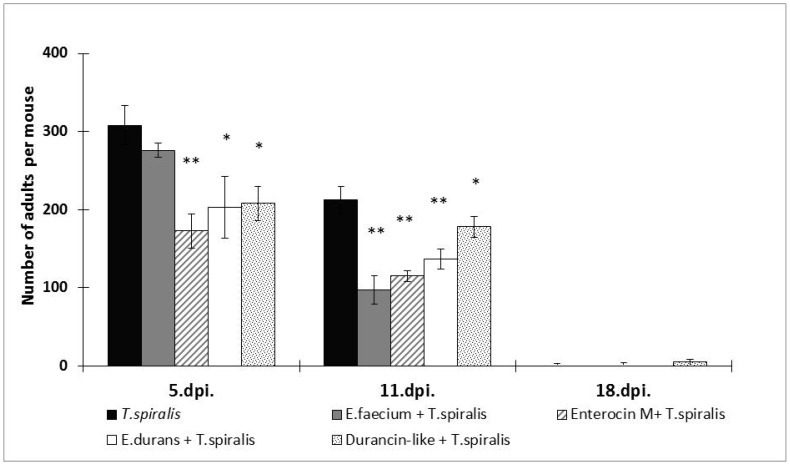
Numbers of adults recovered from mice with enterocins/enterococci treatment and *T. spiralis* infection. * *p* < 0.05; ** *p* < 0.01—significant differences from *T. spiralis* (untreated control).

Both enterocins significantly eliminated the numbers of worms (Figure 2) in the early intestinal phase of trichinellosis on 5 dpi (*p* < 0.01; *p* < 0.05). Enterocin M induced up to a 43.8% reduction in the number of adults compared to its strain *E. faecium* CCM8558, which achieved only 10.4% suppression of parasite infection. The reducing effect of Durancin-like (32.5%) was comparable to its strain *E. durans* ED26E/7 (34.1%). However, in the developed muscle phase at 11 dpi, *E. faecium* CCM8558 most effectively inhibited the presence of worms in the intestine with a reduction of 54.5% (*p* < 0.01). The antiparasitic effect of Enterocin M and *E. durans* ED26E/7 did not change, but the efficacy of Durancin-like decreased to only 16.4%.

**Figure 3:**
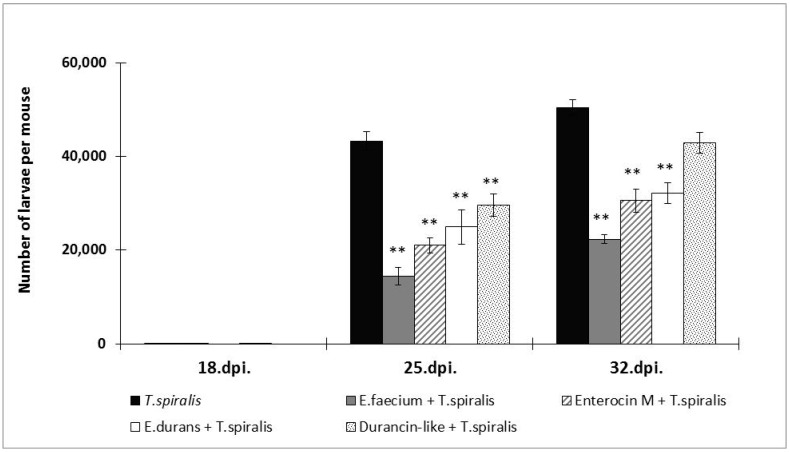
Numbers of muscle larvae recovered from mice with enterocins/enterococci treatment and *T. spiralis* infection. ** *p* < 0.01—significant differences from *T. spiralis* (untreated control).

Enterocins also showed a protective effect in the muscular phase of trichinellosis (Figure 3). The most effective was *E. faecium* CCM8558 (66.5–55.7% reduction). Enterocin M maintained a balanced effect even after the end of NBL migration until the larvae settled in the muscles (51.5–39.6%) and achieved a better result than the strain *E. durans* ED26E/7 (42.4–36.6%). However, Durancin-like reduced its activity from 31.6 to 15%.

Reproductive capacity index (RCI), i.e., the ratio of the number of larvae obtained from one mouse to the number of larvae in the infectious dose/mouse (Table 1), was used as an indicator of parasite infectivity. The RCI in untreated mice reached values (108–126) that were significantly inhibited (*p* < 0.05; *p* < 0.01) in mice with enterocins/enterococci therapy (36–107).

### 3.2. T. spiralis Female’s Fecundity Ex Vivo

**Figure 4:**
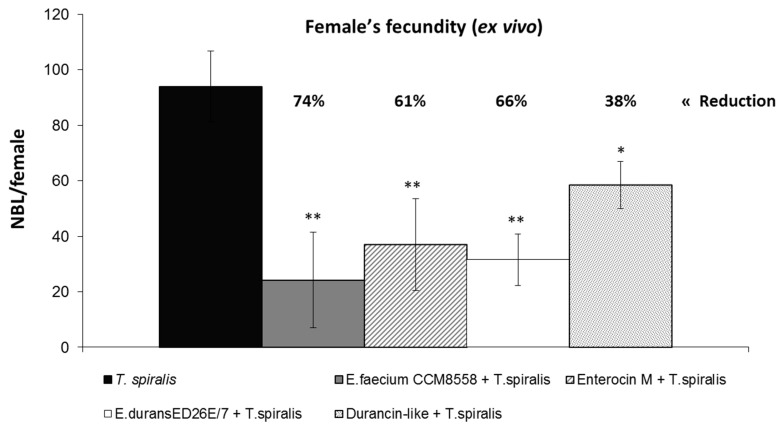
Female’s fecundity ex vivo—numbers of NBL per female isolated from mice with enterocins/enterococci treatment and *T. spiralis* infection. * *p* < 0.05; ** *p* < 0.01—significant differences from *T. spiralis* (untreated control).

The inhibited reproductive activity of *Trichinella* was confirmed in mice after application of enterocins/enterococci (Figure 4). The production of NBL significantly (*p* < 0.05; *p* < 0.01) decreased in mice with the application of both strains and enterocins. *E. faecium* CCM8558 reduced NBL production by 74%, Enterocin M reduced it by 61%, *E. durans* ED26E/7 reduced it by 66%, while Durancin-like had the weakest inhibitory effect at 38%.

### 3.3. T. spiralis Female’s Fecundity In Vitro

**Figure 5:**
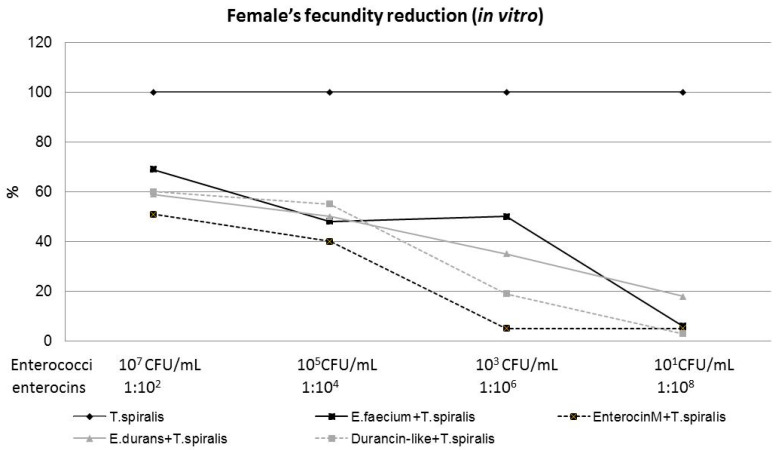
Percentage of NBL reduction in female’s fecundity after cultivation with enterocins/enterococci—the efficacy comparison.

The strain *E. faecium* CCM8558 was the most effective in inhibiting NBL production (Figure 5), compared to *E. durans* ED26E/7 and both enterocins, when it achieved a 50% reduction in female fecundity even at its million-fold dilution. The direct inhibitory action of Enterocin M on NBL production was weak and effective at 51% only at a concentration of 1%. *E. durans* ED26E/7 and Durancin-like influenced female reproduction by the same trend effective up to the concentrations of CFU/mL and 0.01%, respectively. 

**Figure 6:**
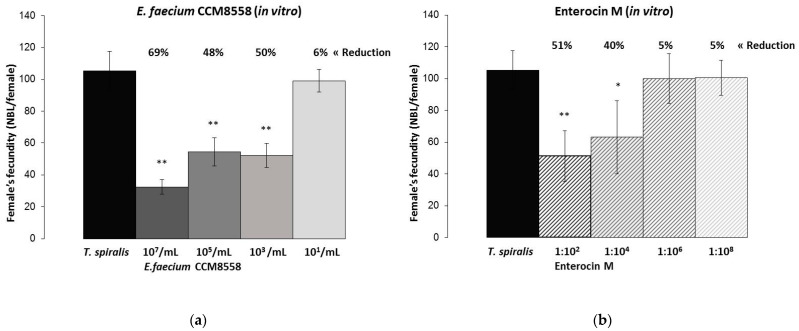

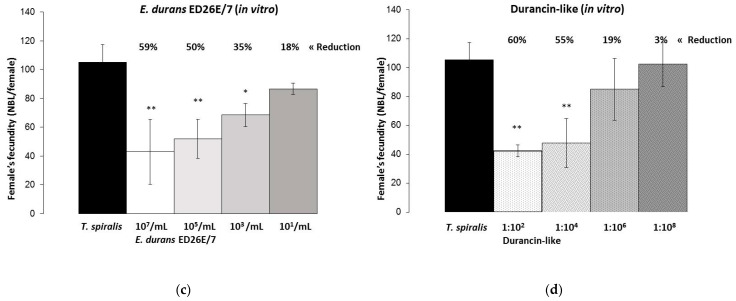
Female’s fecundity in vitro—numbers of NBL per *T. spiralis* female cultivated with enterocins/enterococci: (**a**) *E. faecium* CCM8558 activity against NBL production; (**b**) Enterocin M activity against NBL production.; (**c**) *E. durans ED26E/7* activity against NBL production; (**d**) Durancin-like activity against NBL production. * *p* < 0.05; ** *p* < 0.01—significant differences from *T. spiralis* (untreated control).

The highest reduction in the number of NBL was observed after adding the highest concentration of both enterococci/enterocins to the culture medium (Figure 6). This significant (*p* < 0.05; *p* < 0.01) inhibitory effect was inversely correlated with the strain or enterocin concentration in the medium. *E. faecium* CCM8558 with concentrations of 10^7^, 10^5^, and 10^3^ CFU/mL effectively reduced female fecundity in the range of 69–48%, and Enterocin M with concentrations of 1% and 0.01% inhibited the production of larvae by 40–51%. *E. durans* ED26E/7 with concentrations of 10^7^, 10^5^, and 10^3^ CFU/mL and its enterocin Durancin-like with concentrations of 1% and 0.01% caused comparable efficacy in the reduction of *Trichinella* fertility (59–35%; 55–60%).

## 4. Discussion

Treatment for trichinellosis with conventional anthelmintics is effective only against intestinal worms and not against larvae in muscles. The pathogenicity of *Trichinella spiralis* is determined by the size of the infectious dose and host’s immune status activated by parasitic antigens [33]. The host’s defense against *T. spiralis* is specific in the individual stages of trichinellosis due to the antigenic diversity of the developmental stages of *Trichinella* and the localization of the parasite [34]. Therefore, effective therapy should act comprehensively. Our study examined the anti-*Trichinella* potential of enterocins and their producing strains, which represent an important component of the host gut ecosystem, effective in interactions among microbiota, parasites, and immunity.

Protection against *T. spiralis* infection [35] is proportional to the host’s ability to prevent the development of infective larvae by rapidly eliminating adults from the gut, inhibiting female fecundity, and destroying migrating newborn larvae. Intestinal microbiota plays a key role in the completion of the parasite life cycle and its reproduction [10].

Beneficial properties of enterocins/enterocin-producing strains were recorded during the intestinal phase of trichinellosis. Enterocins/enterococci treatment significantly reduced the worm burden during the first week of infection, when the parasite invades the intestinal epithelium and females start to release NBL into the circulation [36]. Enterocins themselves were also very successful in removing worms; Enterocin M and Durancin-like decreased the adult counts at 5 dpi by 43.8 and 32.5%, respectively. Enterocin M actively controlled the presence of adults in the host even in the developed enteral phase (at 11 dpi) with a 46% reduction, but Durancin-like exerted only 16.4% efficacy. The strain *E. faecium* CCM8558 induced the greatest antiparasitic activity on adults (54.5%) but late in the developed intestinal phase. On the contrary, *E. durans* ED26E/7 maintained a balanced efficacy (34.1 and 35.7%) throughout the intestinal phase. Adhesion of beneficial bacteria to the intestinal epithelium is an important factor for the beneficial action on the host organism. The examined *E. faecium* CCM8558 and *E. durans* ED26E/7 sufficiently colonized the small intestine during *T. spiralis* infection [37]. A similar inhibitory capacity against adult trichinella was recorded in our previous work [12], 53% for *E. faecium* CCM8558 and 38% for *E. durans* ED26E/7. Oral administration of various lactic acid bacteria, *Lactobacillus casei* ATCC 7469 or ATCC 393, *L. plantarum* P164, *L. acidophilus* P110, and *L. paracasei* CNCM, reduced counts of adults in mice by 36–74% [11,14,38]. 

Beneficial probiotic strains were effective in antiparasitic protection because they can modulate their abiotic environment: pH, nutrient content, receptor’s accessibility on epithelial cells, epithelial tight junctions. They also regulate intestinal motility and mucus secretion [9,39], key gut mechanisms of a host’s defense against helminths [40]. The production of bacteriocins and hydrogen peroxide [39,41] from bacteria does not allow parasites to invade the gut epithelial cells, in which molting of trichinella larvae, sexual maturity, and reproduction take place [42].

In the muscle phase of trichinellosis, an increase in the number of muscle larvae was noticed in correlation with the number of adults in the intestine and with the time of the larvae’s migration through the circulation. The highest number of *T. spiralis* larvae was isolated from mice in the control untreated group on 32 dpi. The greatest decrease in the counts of muscle larvae in treated mice was detected at 25 dpi. Enterocin M and Durancin-like showed a moderate lower reduction (51.5 and 31.6%, respectively) in comparison to their producers *E. faecium* CCM8558 and *E. durans* ED26E/7 (66.5 and 42.4%, respectively). Similar inhibitory activities were also detected at the end of the experiment (55.7% for *E. faecium* CCM8558, 39.6% for Enterocin M, 36.3% for *E. durans* ED26E/7) except Durancin-like, which fell to a 15% effect. These results confirmed the significant protective antiparasitic action of Enterocin M itself, which was of comparable intensity to that of its producer *E. faecium* CCM8558. Similar efficacies of strains *E. faecium* CCM8558 and *E. durans* ED26E/7 against *T*. *spiralis* larvae were recorded, with reductions of 65 and 50%, respectively [12]. Application of *Enterococcus faecalis* CECT7121, which did not affect counts of adult *T. spiralis* [15], induced a reduction in the counts of muscle larvae in the range of 33–48%. Lactic acid bacteria *L. casei* ATCC7469, *L. casei* Shirota, *L. plantarum* P164, *L. acidophilus* also showed the beneficial activity in trichinellosis with a reduction in the range of 48–88% [8,11,38,43], but these works used a different experimental design and dosage scheme.

In our study, the infectivity of *T. spiralis* larvae was assessed by the reproductive capacity index (RCI), which reached values of 108–126 in untreated infected mice. It was well above the values detected in mice with enterocins/enterococci treatment (36–107). The reduced reproductive potential of *Trichinella* can be the result of reduced fecundity of females or inhibition of the penetration of newborn larvae into the blood and lymphatic circulation and their subsequent settlement in the striated muscles [12]. We hypothesize that in our work, enterocins/enterococci treatment affected motility and migration of NBL. Enterococci, as well as other LAB, generate secretions (hydrogen peroxide, enterocins, lactic acid, acetic acid), which participate in the damage of pathogens and can also be successful in the host antiparasitic defense [44,45], which resulted in a significant reduction in the larval burden. Bacteriocins together with hydrogen peroxide can affect the viability of larvae or destroy them. Enterocins can probably disrupt the cell membrane of the larval surface structures. Enterocin M and Durancin-like could induce the disruption of the permeability of the larval cuticle and cause the formation of pores in it and thus the leakage of ions, the decrease of ATP, and the bioenergetic collapse of the organism [46].

The relation between the reduced infectious capacity of *T. spiralis* larvae and the reproductive capacity of adult females was verified by testing the fecundity of *Trichinella* females. *T. spiralis* female’s fecundity ex vivo test confirmed a reduced reproductive capacity of *Trichinella* from mice treated with enterocins/enterococci. The inhibitory effects of *E. faecium* CCM8558 and its Enterocin M were higher compared to *E. durans* ED26E/7 and its Durancin-like. *E. faecium* CCM8558 suppressed the production of newborn larvae by 74%, Enterocin M suppressed it by 61%, *E. durans* ED26E/7 suppressed it by 66%, and Durancin-like suppressed it by 38%. Similarly, a suppressive effect of lactobacilli and enterococci on *Trichinella* female fecundity was found [14], despite the fact that the lactobacilli did not affect the number of adults in the gut. Enterococci belong to LAB with production of lactic acid and other organic acids (acetate and butyrate), which [47,48,49] induce low pH in the intestine and can directly affect parasites [50]. The study of El Temsahy [44] confirmed that the acidic gastric pH significantly decreased *T*. *spiralis* female fecundity in both in vivo and in vitro conditions. Authors observed deformations of the uterus, which could disturb embryogenesis, and the consequent inability of females to give birth to newborn larvae.

The direct impact of enterocins/enterococci on parasite reproduction was documented by the *T. spiralis* female fecundity in vitro test. Female’s production of NBL in both ex vivo and in vitro tests were similarly inhibited by examined enterocins/enterococci, except of Durancin-like. The effect of Durancin-like on reducing the number of NBL in vitro was surprisingly high (60–55%), since in other tests, its antiparasitic effect was the weakest. Female fecundity results may not reflect the parasite reproductive capacity in the host organism, where total larval burden represented the lower efficacy of enterocins/enterococci treatment. These differences could be caused by physiological and biochemical processes in the host organism. For example, the jejunum provides a more fertile environment than those in the ileum. This site is more successful for *T. spiralis* reproduction [51]. Another study [42] confirmed that the enteral *T. spiralis* life cycle (along with parasite reproduction) takes place in the intestinal epithelial cells. This efficient host-provided base is missing under in vitro conditions. The interactions of enterocins/enterococci and *Trichinella* fertility was confirmed by our study, but other factors within the host organism [52,53,54,55], such as gut physiology and host immunomodulation with enterocins/enterococci treatment also contributed to the anthelmintic protection. Enterocin M and Durancin-like examined in our study influenced the host’s innate immune response [25] and stimulated blood phagocytes that are effective in destroying *Trichinella spiralis* NBL. Both Enterocin M and Durancin-like increased phagocytosis, as did their producing bacterial strains.

The protective effect of the enterococci/enterocins therapy was manifested by a significant reduction in the parasite burden of the host’s organism; therefore, enterocins as an alternative therapy and prophylaxy for trichinellosis are promising. Several additional mechanisms involved in the anti-parasite defense should be further studied, and the therapeutic use of enterocins should be elucidated. It is unreasonable to suggest enterocins as an alternative to conventional therapies such as anthelmintics, but their use as a complementary therapeutic approach to reduce the risk of infection or to reduce the therapeutic dose of anthelmintics (with many adverse side effects) is more realistic.

## 5. Conclusions

The parasite infectivity and the successful development of the parasitic infection are influenced by four factors: the number of females that reach sexual maturity, their fertility (the number of NBL), the length of time they survive in the intestine, and the length of the viability of muscle larvae [56]. Our results confirmed the anthelmintic effect of selected enterocins and their enterocin-producing strains by accelerating the expulsion of worms from the intestine, (Enterocin M, *E*. *faecium* CCM8558, and *E*. *durans* ED26E/7), by the suppression of female reproduction (both enterocins, both strains), and by the reduction of the muscle larval burden (Enterocin M, *E*. *faecium* CCM8558, and *E*. *durans* ED26E/7). The reduced numbers of *T. spiralis* muscle larvae induced by enterocins or enterococci in our study may be associated with reduced female fecundity or destroying NBL during their migration into host muscles.

All these anti-parasitic mechanisms were acting in cooperation with other host defense mechanisms. The participation of other anti-parasitic mechanisms confirmed differences in obtained results, when low numbers of worms in the gut did not result in low larval burden and vice versa. The protective antiparasitic effect of Enterocin M was comparable to its producing strain *E. faecium* CCM8558 in eliminating adults from the intestine, inhibiting the fertility of *T. spiralis* females and reducing muscle larvae. Durancin-like showed lower efficacy against adult worms or *T. spiralis* larvae in vivo than its producer *E. durans* ED26E/7 but was highly effective in reducing the number of newborn larvae in vitro. Its effect was suppressed in the host organism by host physiology and immunity. 

## Figures and Tables

**Table 1:** Reproductive capacity index of *T. spiralis* from mice with enterocins/enterococci treatment and *T. spiralis* infection.

Days Post Infection	*T. spiralis*	*E. faecium* + *T. spiralis*	Enterocin M + *T. spiralis*	*E. durans* + *T. spiralis*	Durancin-like + *T. spiralis*
25	108.3 ± 4.9	** 36.2 ± 4.6	** 52.5 ± 4.0	** 62.4 ± 9.2	** 74.1 ± 5.8
32	126.3 ± 3.9	** 55.9 ± 2.5	** 76.3 ± 6.2	** 80.4 ± 5.5	107.3 ± 5.7

** *p* < 0.01—statistically significant differences from *T. spiralis* infected group without treatment.

## Data Availability

Data are contained within the article.
